# Authenticity Assessment from Sesame Seeds to Oil and Sesame Products of Various Origin by Differential Scanning Calorimetry

**DOI:** 10.3390/molecules27217496

**Published:** 2022-11-03

**Authors:** Yolanda Victoria Rajagukguk, Mert Atakan Utcu, Mahbuba Islam, Małgorzata Muzolf-Panek, Jolanta Tomaszewska-Gras

**Affiliations:** Department of Food Quality and Safety Management, Poznań University of Life Sciences, Wojska Polskiego 31/33, 60-637 Poznań, Poland

**Keywords:** sesame oil, halva, adulteration, thermodynamic properties, DSC

## Abstract

The aim of this study was to conduct thermal characterization of sesame seeds and oils from various geographical origins (Ethiopia, India, Nigeria, Sudan, Turkey), different method of extraction (hexane and cold-pressing), and different types of derived products (halva and tahini). Thermal characterization was investigated using differential scanning calorimetry (DSC), which showed that origin of the seeds has no influence on the melting profile of sesame oil (peak temperature and enthalpy). Method of extraction (hexane and cold-pressing) influenced the peak temperatures of the resulting oils (*p* ≤ 0.05). The addition of 20% of palm olein to pure sesame oil influenced the significant changes in thermodynamic parameters such as peak temperature (Tm2), which was lowered from −5.89 °C to −4.99 °C, peak half width (T_1/2_), elevated from 3.01 °C to 4.52 °C, and the percentage of first peak area (% peak 1) lowered from 87.9 to 73.2% (*p* ≤ 0.05). The PCA method enabled to distinguish authentic and adulterated sesame oils of various origins. There were no significant differences in thermal properties among the products (halva, tahini) and the authentic sesame oil (*p* > 0.05). The obtained results showed DSC feasibility to characterize sesame oil and sesame products in terms of authenticity.

## 1. Introduction

Sesame (*Sesamum Indicum* L.) is the crop commonly cultivated all over the world mostly in the developing tropical and subtropical areas of Asia, Africa, South, and Central America due to its abundant oilseeds [1]. Sesame seeds contain from 42% to 60% of oil from its weight which is consumed as a flavoring oil and salad dressing in Asia and Europe [2]. Besides utilized for edible oil, sesame seeds were processed to various derived products, such as halva and tahini. Tahini is the paste made of roasted and ground sesame seeds, while halva is sesame paste added with sugar, citric acid, and root extract [3]. Sesame-based products are highly desirable for consumers due to its promising health benefits and bioactive compounds. Sesame seeds are rich in unsaturated fatty acids, vitamin, and antioxidants [4,5,6].

Oils can be extracted from seeds by various methods, for example by pressing, extraction using solvents, or by supercritical fluids. Other alternative attempts to extract better quality of sesame oil were reported in recent years, such as the optimization of screw-pressing at low temperature [7], yield improvement from aqueous extraction [8], and enzyme-assisted aqueous extraction [9]. An extraction method can be considered when it can extract minimally damaged oil, low impurities, high yield, and low amount of waste [10]. Furthermore, the desired final characteristics of oil should also be taken into consideration when choosing the oil extraction method.

Cold pressing technique is commonly used to extract sesame oil because the final product characteristic should be free from chemicals, exhibit natural sensory properties (taste, smell, and color), and safe for direct consumption [7]. Unfortunately, the yield obtained from cold-pressing technique was usually lower than other common oil extraction methods (i.e., solid–liquid and solvent extraction). Lower yield of cold-pressing technique might tempt the profit-driven producer to adulterate or even mislabeled oil from lower quality categories as “cold-pressed” and sell it with high prices. Mislabeling attempt was reported to be one of the most common fraudulent activity in highly-valued oil industry as reported in a recent study [11]. As a countermeasure, rapid thermal characterization to differentiate cold-pressed and hexane-extracted sesame oil can be an important step to face the fraud threat.

Recently, many alerts from EFSA concerned the authenticity of sesame seeds and its derived products [12]. The safety of the sesame products imported from outside Europe was questioned. Due to its high price and high demand, sesame oil is susceptible to be adulterated with lower-priced oils. Detection of adulterated sesame oil was previously studied by several methods such as using Fourier transform infrared (FTIR) combined with chemometrics [13], detection of fatty acid methyl esters by gas chromatography [14], as well as fingerprints determination by ion mobility spectrometry (IMS) [15]. Compared to previously mentioned methods, differential scanning calorimetry (DSC) offer convenient and rapid method of detection, without the utilization of hazardous chemical and solvent. DSC can measure the amount of heat absorbed and released by food components, such as fat, protein, and carbohydrate. Detection of adulterant in oil using DSC is conducted based on the unique characteristics of fatty acid and triacylglycerol composition [16].

In contrast to the feasibility of DSC, the knowledge related to DSC application to analyze other edible oils than olive oils are still limited. Therefore, the aim of this present study was to conduct thermal characterization of sesame seeds and oils from (a) various geographical origins, (b) different method of extraction i.e., cold-pressing and hexane extraction, and (c) different types of derived products i.e., halva and tahini. Sesame seeds originated from Ethiopia, India, Nigeria, Sudan, and Turkey were investigated for the DSC analysis. The susceptibility of changes in DSC melting profile were investigated by the addition of 20% palm olein to sesame seed oil. Palm olein was chosen as the adulterant due to its frequent utilization in food fraud report and authenticity study [17]. Additionally, palm oil is known as one of the cheapest edible oil that costs 2–3 times lower than sesame oil. The melting profiles of oils extracted from various products such as tahini and halva were also compared to thermal properties of pure sesame seed oil for authenticity determination.

## 2. Results and Discussion

Samples analyzed during the experiments consisted of: (a) sesame seeds from five geographical origins (Ethiopia, India, Nigeria, Sudan and Turkey), (b) sesame oils extracted from seeds by hexane and cold-pressing, (c) sesame-derived products, such as halva (original, added with cocoa, and added with pistachio) and tahini. Prior to the extraction, the moisture content analysis of sesame seed was conducted. The oil yield obtained from each extraction was calculated. The characteristic of the resulting oils were then analyzed based on the fatty acid composition and DSC melting profile (peak temperature and enthalpy).

### 2.1. Moisture Content of Seeds and Yield

Moisture content of sesame seeds is presented in Table 1. There are no significant differences in moisture content among sesame seeds from various geographical origin (*p* > 0.05). Water content is an important quality feature of seeds because it could affect the seed longevity during storage [18]. High water content could influence rapid deterioration of seeds caused by microbial growth and chemical changes [19].

**Figure 1:**
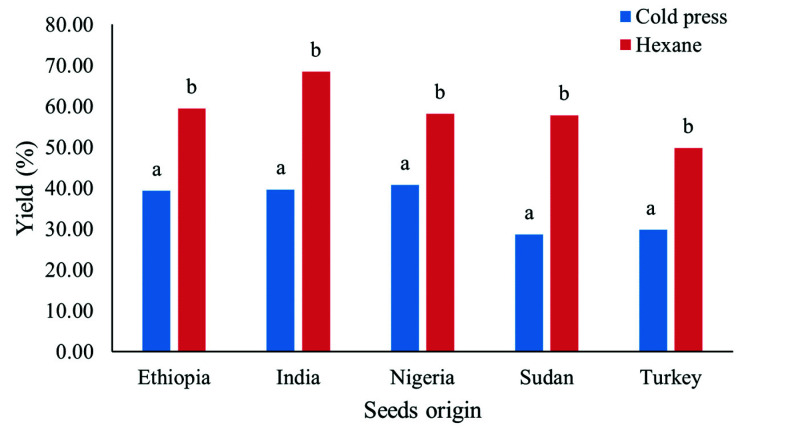
Yield of oil from hexane extraction and cold pressing of sesame seeds with various origin. Different letters (a, b) indicate significant differences between mean values (*p* ≤ 0.05).

Bhuiya et al. [20] mentioned that moisture content can influence the percentage of yield obtained from the extraction. The authors mentioned that the decreased moisture content of the seed affected the physical appearance of oil, it resulted in higher oil residue and cakey appearance. Yield of hexane extraction and cold pressing was depicted in Figure 1. The results showed that higher yield of sesame oils was obtained by oil extraction compared to cold pressing (*p* ≤ 0.05). Similar results were also reported by Bhuiya et al. in the comparative study on the yield of beauty leaf seeds oil obtained from n-hexane and cold-pressed extraction [20]. The authors concluded that the n-hexane extraction technique was more efficient than screw press technique.

### 2.2. Fatty Acid Composition

To establish the content of dominant fatty acids in sesame oils and sesame products, analysis of fatty acid composition (FA) was conducted. Fatty acid composition of sesame oils from various origin and sesame products (halva, tahini) is presented in Table 2. The results showed that the major components of fatty acids in sesame oil are linoleic (C18:2), oleic (C18:1), stearic (C18:0), and palmitic (C16:0) acid. The concentration of linoleic acid varied from 40.56 to 44.62%, the second most abundant fatty acid is oleic acid that ranged from 39.88 to 43.31%. The FA composition is consistent with the Codex Alimentarius standard [21], which established the range of linoleic acid in sesame oil was between 41.5–47.9% and 35.9–42.3% for oleic acid. Similar amount of FA content in sesame oils was also reported in another study [5], the author reported the FA content in sesame oil consist of oleic acids (36.13–43.63%), linoleic acid (39.13–46.38%), palmitic acid (8.19–10.26%), stearic acid (4.63–6.35%).

Sesame oils extracted from seeds of various geographical origins differed in the highest content of certain FA components. For example, sesame oil extracted from Turkish sesame seeds contained highest amount of oleic and linoleic acid, while Ethiopian sesame seeds had higher palmitic and stearic acid. According to Kurt [5], factors affecting the amount of fatty acid in the samples are: genotype, location, temperature, condition, moisture content, growing condition, planting date, as well as fertilization of the seeds.

In the case of halva, one of the sesame products, lower amount of linoleic and oleic acids was observed, especially the ones with cocoa and pistachio addition (HC and HP samples). Additionally, the content of linoleic acid in sesame products (HC, HP) was below the limits established by Codex Alimentarius standard [21]. Sesame oils were reported to contain higher percentage of linoleic and oleic fatty acids among other vegetables oils, significant changes of those fatty acid percentages may became the key of authenticity determination [14]. Lower percentage of linoleic acid in sesame products (HC, HP, T) might be affected by the heat treatment during processing. The results were in agreement with Hama [22] that reported a significant decrease in linoleic acid in sesame oil extracted from roasted sesame seeds. Following the decreased amount of unsaturated fatty acid (18:1, 18:2), ratio of the unsaturated fatty acid (UFA) to saturated fatty acid (SFA) was calculated. Ratio of UFA to SFA is often used as an index to measure the edible oil quality from nutritional approach [23]. The lowest UFA/SFA ratio was observed in oil extracted from halva with cocoa (3.0), while the highest ratio was observed in Turkish and Nigerian sesame seed oil (5.8).

### 2.3. Thermodynamic Properties

DSC technique was applied to analyze thermal properties of: (a) sesame seeds from five geographical origins (Ethiopia, India, Nigeria, Sudan and Turkey), (b) sesame oils extracted from seeds by hexane (HE) and cold-pressing (CP), (c) authentic and adulterated sesame oils, and (d) sesame-derived products (halva and tahini).

#### 2.3.1. Effect of Different Methods of Extraction and Geographical Origins on Thermal Properties of Sesame Oil

Melting peak temperature (Tm) and enthalpy (∆Hm) were calculated from the heating curves of sesame seeds and sesame oils obtained by hexane extraction (HE) and cold pressing (CP) (Table 3). Generally, seeds origin does not significantly influence the Tm of all seeds and oils samples (*p* > 0.05). However, the enthalpies of sesame oil CP were able to differentiate the oils based on the seed origins (*p* ≤ 0.05). Besides the seed’s origin, general comparison of sesame seeds and sesame oils (HE, CP) based on Tm and ∆Hm were also conducted. The results showed no significant differences observed in the Tm and ∆Hm of sesame seeds and oils (HE, CP) (*p* > 0.05), except in Sudanese sesame. Following the results, it is worth to mention that sesame was able to exhibit constant thermal characteristic despite being tested in the form of seeds or oils.

**Figure 2:**
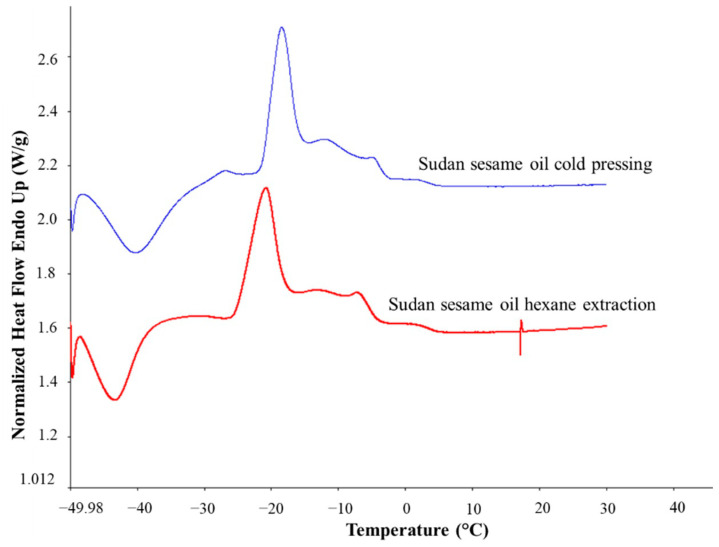
DSC melting curves of sesame oils obtained from extraction by hexane and cold-pressing.

Concerning the thermal characteristics of HE and CP oils, lower average value of ∆Hm was observed in CP oils. Additionally, average Tm of sesame oil CP was significantly higher than sesame oil HE (*p* ≤ 0.05). Melting curves comparison between sesame oil extracted by hexane and cold press extraction are depicted in Figure 2. The results indicated that oils obtained from different extraction method may contain different compositional properties. Study on the thermal properties of cold pressed versus solvent extracted lemon seed oil reported that four melting peaks in cold press lemon seed oil were observed, while only three peaks for solvent extracted oil [10]. The authors also mentioned different percentage of sterol and tocopherol composition found in each extracted oil. Another supporting finding was also reported by Stamenković et al. [24], oil obtained from Soxhlet or hexane extraction contained higher percentage of saturated fatty acid compared to seed oil obtained from cold press extraction.

Summing up, the general results showed that geographical origins does not significantly influenced peak temperature of sesame seeds and oils (CP and HE) (*p* > 0.05). While method of extraction was showed to have influence on the average melting peak temperature (Tm) of sesame oils.

#### 2.3.2. Effect of Adulteration of Sesame Oil with Palm Olein on Thermal Properties

**Figure 3:**
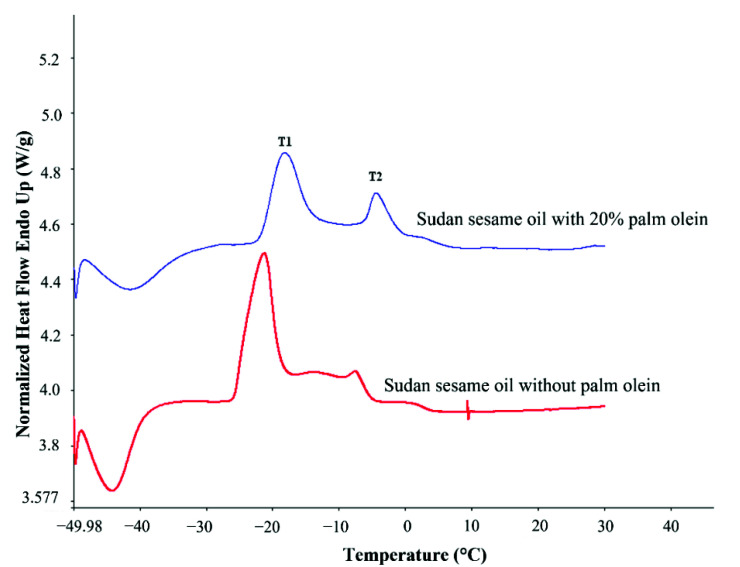
DSC melting curves of authentic and adulterated sesame oil.

Thermodynamic properties determined from the DSC melting curves of pure and adulterated sesame oils are presented in Table 4 and Table 5. From the melting curve of pure sesame oil, two main peaks were detected (Figure 3) at average temperatures of −19.93 °C and −5.89 °C (Table 4). Temperature values for both peaks of pure sesame oils were consistent with those given by Tan and Che Man [25], where first peak was detected at temperature of −21.45 °C and second peak at −9.62 °C. These peaks shifted after the addition of 20% palm olein to higher temperature values of −19.26 °C and −4.99 °C, respectively.

Significant differences between peak temperatures of pure and adulterated oils were observed especially for the second peak (Tm2) of sesame oil from Nigeria and Sudan (*p* ≤ 0.05). Similar results of Tm2 increment in melting curve was also reported in another study of sesame oil adulteration with palm stearin [26]. The differences in peak temperature are due to the change in the saturated to unsaturated acid ratio, because it is known that palm olein contains higher amount of saturated fatty acids (49.67%) compared to sesame oil [27].

The results presented in Table 5, showed that there were no significant differences in total enthalpy of melting transition (∆Hm total) between pure and adulterated sesame oil (*p* > 0.05), except sesame oil from Nigeria. However, there were significant differences in the enthalpy of melting transition of the first (∆Hm Peak 1) and second peak (∆Hm Peak 2), as well as for the percentage of ∆Hm for the first peak (% peak 1) (*p* ≤ 0.05) (Table 5). The changes in the shape of both peaks can be also expressed by another parameter i.e., peak half width (T_1/2_), for which significant differences were observed between pure sesame oil (3.01 °C) and adulterated sesame oil (4.52 °C) (*p* ≤ 0.05).

#### 2.3.3. Thermal Properties of Sesame Oils Extracted from Different Types of Sesame Products

Thermodynamic parameters (enthalpy, peak temperature, peak half width) of sesame oils HE (mean values) and oils extracted from sesame products i.e., halva (H), halva cocoa (HC), halva pistachio (HP), and tahini (TH) are presented in Table 6. Despite being extracted from several types of sesame products, the results showed that all of thermodynamic properties of the samples did not differ significantly (*p* > 0.05). The results indicate the applicability of DSC thermodynamic parameters to determine the authenticity of sesame oil and its derived products.

**Figure 4:**
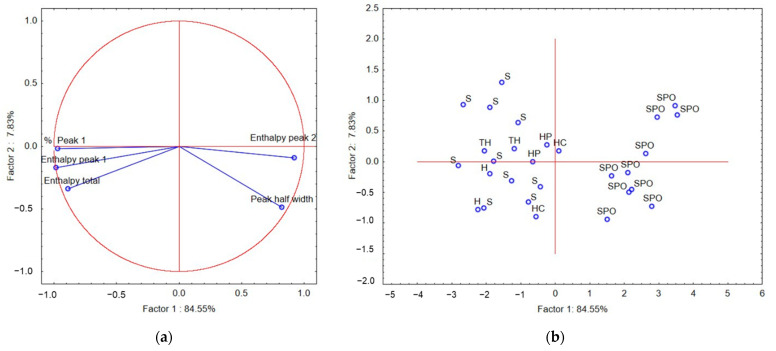
The loading (**a**) and scatter plots (**b**) for PC1 and PC2 analyses of DSC thermodynamic parameters of first heating (% Peak 1, ∆Hm Peak 1, ∆Hm Peak 2, ∆Hm total, and peak half width) in sesame products (H, HC, HP, TH), authentic (S) and adulterated (SPO) sesame oils. S: sesame oil, SPO: sesame oil adulterated with palm olein, H: halva, HC: halva cocoa, HP: halva pistachio, TH: tahini.

The relation among thermodynamic parameters (% Peak1, ∆Hm Peak1, ∆Hm Peak2, ∆Hm total, and T_1/2_), as well as scatter plots of sesame products (H, HC, HP, TH), authentic (S) and adulterated (SPO) sesame oils are depicted in Figure 4a,b respectively. Similar study about the implementation of PCA as an authenticity assessment was reported by Tomaszewska-Gras [28]. Figure 4a visualizes the relations between two first principal components (PC1 and PC2), which described 92.38% of the initial variability. PC1 explains the observed variability in 84.55% and PC2 in 7.83%, which indicates that almost all variables are highly correlated with PC1. In the case of parameter of peak half width (T_1/2_) there was also correlation to PC2. Additionally, variables such as ∆Hm Peak 1, ∆Hm total, and % Peak1 are positively correlated with each other, and negatively correlated with the rest of variables (∆Hm Peak 2 and T_1/2_). The observation explains the data presented in Table 5 and Table 6, the greater value of i.e., ∆Hm Peak1, the lower value of ∆Hm Peak2. PCA Scatter plots of DSC thermodynamic parameters of sesame products (H, HC, HP, TH), authentic (S) and adulterated (SPO) sesame oils are presented in Figure 4b. The results show visible distinction between pure and adulterated samples. The objects were separated in two clusters. Pure sesame oils and products were located on the negative side of the *X*-axis, while adulterated samples were placed on the positive side of the *X*-axis.

## 3. Materials and Methods

### 3.1. Materials

The material used for the experiments composed of sesame seeds obtained from five different geographical origin (Ethiopia Humera district, India, Nigeria Masduguri district, Sudan Blue Nile district, and Turkey Egean district), three samples of halva (original halva, halva with pistachio, and halva with cocoa), and one sample of tahini. Indian sesame seeds were purchased from Helio S.A (Brochów, Poland), while the rest of sesame seeds samples were obtained from Ari Susam (İstanbul, Turkey). All halva samples were produced by Şarkütire (İzmir, Turkey) and tahini was produced by Koska (İstanbul, Turkey).

Prior to extraction, sesame seeds were sieved using automatic sieving machine (Multiserw, Brzeżnica, Poland) with several diameter of sieves (0.7 mm, 1.0 mm, and 3.0 mm). For the experiment with adulteration of sesame oil, palm olein 20% (*w*/*w*) was added to each sesame oil. The samples were mixed and stored at 4 °C for 2–3 days.

### 3.2. Methods

#### 3.2.1. Oil Extraction from Sesame Seeds

A total of 50 g from each sample of sesame seeds, halva, and tahini was weighed. The samples were then blended for 2 min, followed by hexane addition with 1:3 (*w*/*v*) ratio, then were transferred to an automatic stirrer (MS-H-Pro plus, Chemland, Poland) and constantly stirred under 0.1× *g* for 60 min. After extraction, samples were centrifuged (MPW-380R, MPW Med. Instruments, Warsaw, Poland) at 112,000× *g* for 20 min, then the supernatants were collected and transferred to the flask of vacuum rotary evaporator (Heidolph laborota 4003, Darmstadt, Germany). Evaporation was conducted under 330 mbar at 40–45 °C. The remaining hexane was removed by exposing the oils with nitrogen gas for 5 min. The resulting oils were proceeded to further analysis. Yield obtained from the extraction was calculated.

#### 3.2.2. Cold-Pressing

Sesame seeds (150 g) were weighed and pressed at 40 °C using oil presser (Dulong DL-ZYJ06, Jiangxi, China). The resulting oils were kept in light resistant brown glass bottles and stored in a refrigerator at −20 °C until the analysis took place.

#### 3.2.3. Moisture Analysis

The samples (2 g) were placed into a moisture analyzer (MA150 Sartorius Lab Instruments, Goettingen, Germany). Two replications of analysis were performed on each sample. Moisture content was calculated by subtracting 100% with the percentage of dry matter (DM). Dry matter was calculated as follows:

DM (%) = [dry seeds weight (g)/seed weight (g)] × 100,(1)

#### 3.2.4. Differential Scanning Calorimetry (DSC)

A Perkin Elmer DSC 7 differential scanning calorimeter (Perkin Elmer, Norwalk, CT, USA) equipped with an Intracooler II and running under Pyris software was used to examine the melting properties of sesame oils, seeds, and sesame products. Nitrogen (99.999% purity) was the purge gas. The DSC calorimeter was calibrated using indium (m.p. 156.6 °C, ∆Hf = 28.45 J/g) and n-dodecane (m.p. −9.65 °C, ∆Hf = 216.73 J/g). Samples of sesame oil, grounded seeds, and sesame products (8–10 mg) were weighed into aluminum pans of 20 μL (Perkin Elmer, Norwalk, CT, USA No. 0219-0062) and hermetically sealed. The reference was an empty, hermetically sealed aluminum pan. The sample pan was placed in the calorimeter at 10 °C were analyzed according to the temperature program: (1) cooling from 10 °C to −50 °C at 20 °C/min; (2) isotherm at −50 °C for 1 min; (3) heating from −50 °C to 30 °C at 5 °C/min; (4) isotherm 1 min at 30 °C; (5) cooling from 30 °C to −50 °C at 5 °C/min; (6) isotherm for 1 min at −50 °C; (7) heating from −50 °C to 30 °C at 5 °C/min. Two replicates were analyzed for each sample.

The following parameters were analyzed from the melting DSC curves: peak temperature (T), enthalpy of melting (∆H (J/g), determined as the area limited by the melting curve and the base line. Determination of temperature and enthalpy of melting in sesame oil and seeds samples was performed according to standard ISO 11357-3:2011 [29].

#### 3.2.5. Determination of Fatty Acid Composition Using GC

Fatty acid composition of sesame oils extracted from seeds of various origin and sesame products (halva, tahini) were determined by gas chromatography. The analysis of fatty acids composition was based on the AOCS official method CE 2-66 [30]. Two drops of fat were dissolved in 1 mL of hexane (for HPLC, Sigma Aldrich, St. Louis, MO, USA), followed by the addition of 1 mL of 0.4 N sodium methoxide. Samples were mixed for 15 min, then 5 mL of distilled water was added after and the upper layer was taken. Fatty acid methyl esters were analyzed using a Trace 1300 chromatograph (Thermo Fisher Scientific, Waltham, MA, USA). Separation was performed on a Supelcowax 10 capillary column (30 m × 0.2 mm × 0.2 µm). The injection was performed in spitless mode. The sample volume was 1 µL and hydrogen was used as the carrier gas. The initial furnace temperature was 160 °C and was increased from 12 °C/min to 220 °C. A temperature of 220 °C was maintained for 20 min. Fatty acid methyl esters were identified based on comparing the retention times in the sample and in the 37-Component FAME Mix (Supelco).

#### 3.2.6. Statistics

Means and standard deviation were calculated using Tibco Statistica 13.3 software (Tibco Software Inc., Tulsa, OK, USA). Statistical analysis ANOVA and Tukey’s honest significant difference test (*p* ≤ 0.05) were performed to identify the differences between groups. Principal component analysis (PCA) was performed to distinguish pure sesame oils from the adulterated oils based on the data from DSC thermodynamic parameters (enthalpies, peak half width).

## 4. Conclusions

Presented DSC study showed that the origin of sesame seeds did not significantly influence thermal properties of sesame oils i.e., peak temperature and enthalpy of melting phase transition. Comparison of two methods of extraction (by hexane and cold pressing) revealed that the melting peak temperatures differed significantly in contrast to the enthalpy. The addition of 20% of palm olein to sesame oil resulted in the changes of the melting curve shape and changes in the melting transition parameters. It was manifested by a decrease in the first peak area (i.e., ∆Hm Peak1 and % peak), in favor of the growth of the second peak area (∆Hm Peak2). Significant changes in the second peak temperature (T2) and peak half width (T_1/2_), as well as enthalpy (∆Hm Peak2) were stated. PCA method enabled to distinguish authentic and adulterated sesame oils of various origins. In this study thermal properties of sesame-derived products (halva and tahini) were also analyzed. It was reported that there were no significant differences between the products and the authentic sesame oil. Considering the obtained results, DSC technique was proven to have a potential as an analytical technique to characterize sesame oil and sesame products.

## Figures and Tables

**Table 1:** Moisture content of sesame seeds from different origin.

Seeds Origin	Moisture (%)
Ethiopia	6.11 ± 0.98 a
India	5.83 ± 0.45 a
Nigeria	5.18 ± 0.04 a
Sudan	5.25 ± 0.50 a
Turkey	4.90 ± 0.45 a

Values are given as mean ± SD (n = 2). The same lowercase letters (a) indicate no significant differences (*p* > 0.05).

**Table 2:** Fatty acid content of sesame oils from different origin and sesame products.

Fatty Acid	Sesame Oils (%)					Sesame Products (%)			
	SO Ethiopia	SO India	SO Nigeria	SO Sudan	SO Turkey	H	HC	HP	T
C16:0	9.61	9.33	8.98	9.38	8.62	12.53	15.57	13.91	9.25
C16:1	0.08	0.09	0.09	0.08	0.06	0.05	0.07	0.09	0.06
C18:0	6.09	4.95	4.91	5.52	5.23	7.16	8.87	7.64	5.64
C18:1	42.70	39.88	40.58	42.79	43.31	37.59	38.63	38.79	42.05
C18:2	40.56	44.62	44.16	41.00	41.54	41.43	35.81	38.31	41.96
C18:3(n-3)	0.18	0.20	0.32	0.21	0.33	0.24	0.19	0.22	0.25
C20:0	0.72	0.85	0.86	0.91	0.83	0.81	0.62	0.80	0.69
C20:1	0.06	0.08	0.10	0.10	0.10	0.09	0.09	0.09	0.10
ƩSFA	16.42	15.13	14.75	15.82	14.68	20.58	25.20	22.50	15.58
ƩMUFA	42.84	40.05	40.77	42.97	43.47	37.73	38.79	38.97	42.21
ƩPUFA	40.74	44.82	44.48	41.21	41.87	41.67	36.00	38.53	42.21
UFA/SFA	5.1	5.6	5.8	5.3	5.8	3.9	3.0	3.4	5.4

SO: sesame oil, H: halva, HC: halva with cocoa, HP: halva with pistachio, T: tahini, ƩUFA: total unsaturated fatty acids, ƩSFA: total saturated fatty acids, ƩMUFA: total monounsaturated fatty acids, ƩPUFA: total polyunsaturated fatty acids.

**Table 3:** DSC enthalpy and peak temperature determined from heating curve of sesame seed and sesame oils obtained from extraction by hexane (HE) and cold pressing (CP).

Seeds Origin	Sesame Seed	Sesame Oil (HE)		Sesame Oil (CP)	
	Peak Temperature	Enthalpy	Peak Temperature	Enthalpy	Peak Temperature
	Tm1 (°C)	∆Hm (J/g)	Tm1 (°C)	∆Hm (J/g)	Tm1 (°C)
Ethiopia	−18.40 ± 0.64 ^aA^	42.36 ± 3.45 ^aA^	−20.25 ± 1.16 ^aA^	39.48 ± 0.50 ^aBC^	−19.20 ± 0.81 ^aA^
India	−19.12 ± 0.79 ^aA^	41.14 ± 3.77 ^aA^	−20.07 ± 0.02 ^aA^	33.94 ± 0.23 ^aA^	−19.30 ± 0.38 ^aA^
Nigeria	−19.63 ± 0.39 ^aA^	40.75 ± 0.46 ^aA^	−19.25 ± 0.32 ^aA^	39.61 ± 0.60 ^aBC^	−19.24 ± 0.62 ^aA^
Sudan	−19.10 ± 0.88 ^bA^	42.64 ± 1.10 ^bA^	−21.00 ± 0.35 ^aA^	35.69 ± 2.26 ^aAB^	−18.64 ± 0.14 ^bA^
Turkey	−19.68 ± 0.93 ^aA^	43.80 ± 0.46 ^aA^	−19.41 ± 0.20 ^aA^	41.88 ± 1.30 ^aC^	−18.89 ± 0.34 ^aA^
Mean	−19.18 ± 0.75 ^ab^	42.01 ± 2.04 ^a^	−19.93 ± 0.78 ^a^	37.90 ± 3.18 ^a^	−19.02 ± 0.46 ^b^

HE: hexane extraction. CP: cold pressed. Values are given as mean ± SD (*n* = 2). Different lowercase letters (a, b) in the same row under the same thermodynamic property indicate significant difference (*p* ≤ 0.05). Different uppercase letters (A,B,C, in the same column indicate significant difference (*p* ≤ 0.05).

**Table 4:** DSC parameters of melting transition consist of temperature (Tm) and peak half width (T_1/2_) in authentic and adulterated sesame oils.

Seeds Origin	Sesame Oils			Sesame Oils + 20% Palm Olein		
	Tm1 (°C)	Tm2 (°C)	T_1/2_ (°C)	Tm1 (°C)	Tm2 (°C)	T_1/2_ (°C)
Ethiopia	−20.25 ± 1.16 ^a^	−6.35 ± 1.02 ^a^	3.75 ± 0.62 ^a^	−19.14 ± 0.02 ^a^	−5.1 ± 1.01 ^a^	4.72 ± 0.27 ^a^
India	−20.07 ± 0.02 ^a^	−5.11 ± 0.51 ^a^	2.75 ± 0.15 ^a^	−20.69 ± 0.37 ^a^	−5.51 ± 0.53 ^a^	4.36 ± 0.27 ^b^
Nigeria	−19.25 ± 0.32 ^a^	−5.37 ± 0.09 ^a^	2.71 ± 0.14 ^a^	−18.29 ± 0.33 ^b^	−3.92 ± 0.21 ^b^	4.17 ± 0.04 ^b^
Sudan	−21.00 ± 0.35 ^a^	−7.13 ± 0.39 ^a^	2.91 ± 1.46 ^a^	−18.88 ± 0.98 ^a^	−4.87 ± 0.57 ^b^	4.96 ± 0.46 ^a^
Turkey	−19.41 ± 0.20 ^a^	−5.49 ± 0.01 ^a^	2.95 ± 0.29 ^a^	−19.33 ± 0.00 ^a^	−5.58 ± 0.49 ^a^	4.40 ± 0.05 ^b^
Mean	−19.93 ± 0.78 ^a^	−5.89 ± 0.88 ^a^	3.01 ± 0.68 ^a^	−19.26 ± 0.92 ^a^	−4.99 ± 0.78 ^b^	4.52 ± 0.36 ^b^

Values are given as mean ± SD (*n* = 2). Different letters (a, b) in the same row under the same thermodynamic property indicate significant difference (*p* ≤ 0.05).

**Table 5:** DSC parameters of melting transition consist of total and partial enthalpy in authentic and adulterated sesame oils.

Seeds Origin	Sesame Oils				Sesame Oils + 20% Palm Olein			
	∆Hm Total (J/g)	∆Hm Peak1 (J/g)	∆Hm Peak2 (J/g)	% Peak1	∆Hm Total (J/g)	∆Hm Peak1 (J/g)	∆Hm Peak2 (J/g)	% Peak1
Ethiopia	42.36 ± 3.45 ^a^	37.07 ± 4.08 ^a^	5.29 ± 0.63 ^a^	87.41 ± 2.50 ^b^	36.35 ± 0.90 ^a^	27.20 ± 1.43 ^a^	9.15 ± 0.53 ^b^	74.80 ± 2.09 ^a^
India	41.14 ± 3.77 ^a^	37.57 ± 3.97 ^a^	3.57 ± 0.20 ^a^	91.26 ± 1.29 ^b^	39.79 ± 1.12 ^a^	30.05 ± 0.94 ^a^	9.74 ± 0.17 ^b^	75.53 ± 0.25 ^a^
Nigeria	40.75 ± 0.46 ^b^	35.66 ± 1.43 ^b^	4.96 ± 0.86 ^a^	87.83 ± 4.74 ^b^	34.13 ± 0.45 ^a^	23.86 ± 1.02 ^a^	10.27 ± 0.57 ^b^	69.89 ± 2.08 ^a^
Sudan	42.64 ± 1.10 ^a^	37.35 ± 1.99 ^b^	5.29 ± 0.89 ^a^	87.57 ± 2.40 ^b^	34.43 ± 2.86 ^a^	24.92 ± 2.81 ^a^	9.50 ± 0.05 ^b^	72.31 ± 2.15 ^a^
Turkey	43.80 ± 0.46 ^b^	37.42 ± 0.33 ^b^	6.39 ± 0.78 ^a^	85.42 ± 0.14 ^b^	38.33 ± 1.12 ^a^	28.16 ± 0.22 ^a^	10.17 ± 0.90 ^b^	73.49 ± 1.57 ^a^
Mean	42.01 ± 2.04 ^a^	37.02 ± 2.19 ^b^	5.10 ± 1.09 ^a^	87.90 ± 2.82 ^b^	36.60 ± 2.58 ^a^	26.84 ± 2.61 ^a^	9.77 ± 0.59 ^b^	73.20 ± 2.48 ^a^

Values are given as mean ± SD (*n* = 2). Different letters (a, b) in the same row under the same thermodynamic property indicate significant difference (*p* ≤ 0.05).

**Table 6:** DSC peak temperatures (Tm), enthalpy (∆Hm), and peak half width (T_½_) of sesame products.

Sesame Products	∆Hm Total (J/g)	∆Hm Peak1 (J/g)	∆Hm Peak2 (J/g)	% Peak1	Tm1 (°C)	T_1/2_ (°C)
SO-HE	42.11 ± 2.13 ^a^	37.02 ± 4.08 ^a^	5.10 ± 1.09 ^a^	87.90 ± 2.82 ^a^	−20.25 ± 1.16 ^a^	3.01 ± 0.68 ^a^
H	42.40 ± 1.11 ^a^	38.88 ± 1.29 ^a^	3.52 ± 0.18 ^a^	91.69 ± 0.64 ^a^	−20.52 ± 0.35 ^a^	3.64 ± 0.19 ^a^
HC	39.49 ± 2.46 ^a^	36.73 ± 2.61 ^a^	5.17 ± 1.37 ^a^	87.63 ± 3.18 ^a^	−19.84 ± 0.57 ^a^	3.59 ± 0.58 ^a^
HP	39.57 ± 1.17 ^a^	33.81 ± 1.12 ^a^	5.75 ± 0.05 ^a^	85.45 ± 0.30 ^a^	−18.90 ± 0.07 ^a^	3.63 ± 0.06 ^a^
TH	42.86 ± 0.51 ^a^	37.08 ± 1.58 ^a^	5.78 ± 1.07 ^a^	86.50 ± 2.66 ^a^	−19.72 ± 0.21 ^a^	2.80 ± 0.09 ^a^

SO-HE: sesame oil from hexane extraction, H: halva, HC: halva cocoa, HP: halva pistachio, TH: tahini. Values are given as mean ± SD (*n* = 2). Different letters (a, in the same column indicate significant difference (*p* ≤ 0.05).

## Data Availability

The data used to support the findings of this study can be made available by the corresponding author upon request.
